# Asystole Following Jaw Thrust Maneuver: A Case Report

**DOI:** 10.7759/cureus.71077

**Published:** 2024-10-08

**Authors:** Jingxia Meng, Yonggang Wang, Mira Kehar, Lazar Popilevsky

**Affiliations:** 1 Anesthesiology, Metropolitan Hospital, New York, USA

**Keywords:** asystole, bradycardia, jaw-thrust maneuver, pain, parasympathetic, vasovagal

## Abstract

The jaw thrust maneuver is a fundamental airway management tool to prevent the tongue from obstructing the upper airway in unconscious patients. Known complications of the jaw thrust maneuver include spinal cord injury if the cervical spine is unstable and exacerbation of an existing mandibular injury. However, this procedure is frequently associated with pain, and the associated consequences, especially the parasympathetic response, are rarely seen or discussed. We report a rare complication of a 38-year-old healthy female emerging from general anesthesia who developed transient but severe bradycardia leading to asystole following a jaw thrust maneuver. We conclude that the bradycardia and asystole resulted from the vagal response to the pain induced by the jaw thrust.

## Introduction

Airway obstruction associated with general anesthesia is generally attributed to reduced genioglossus activity and consequent posterior placement of the tongue. Maintenance of the airway patency is of paramount importance for anesthesiologists. A vigorous jaw thrust focuses on displacing the jaw forward to establish an open airway. Commonly known complications include sympathetic response, bruising, and worsening cervical spine injury [1]. This procedure is associated with certain pain, and pain is a known trigger for vasovagal syncope [2]. However, the adverse effect of this pain involving parasympathetic response is rarely seen or discussed. Especially when a patient is under general anesthesia, prodromal symptoms of severe pain can be obscured, with the only initial presentation being the sudden onset of bradycardia or even asystole. We present the case of a healthy female emerging from general anesthesia who developed transient asystole following a jaw thrust maneuver.

## Case presentation

A 38-year-old female with American Society of Anesthesiologists (ASA) physical status class 1 (height 165 cm, weight 75 kg, BMI 27) and a history of abnormal uterine bleeding presented for hysteroscopy, excision tissue, and dilation and curettage. No significant past medical history was noted on chart review and pre-anesthesia evaluation.

**Figure 1:**
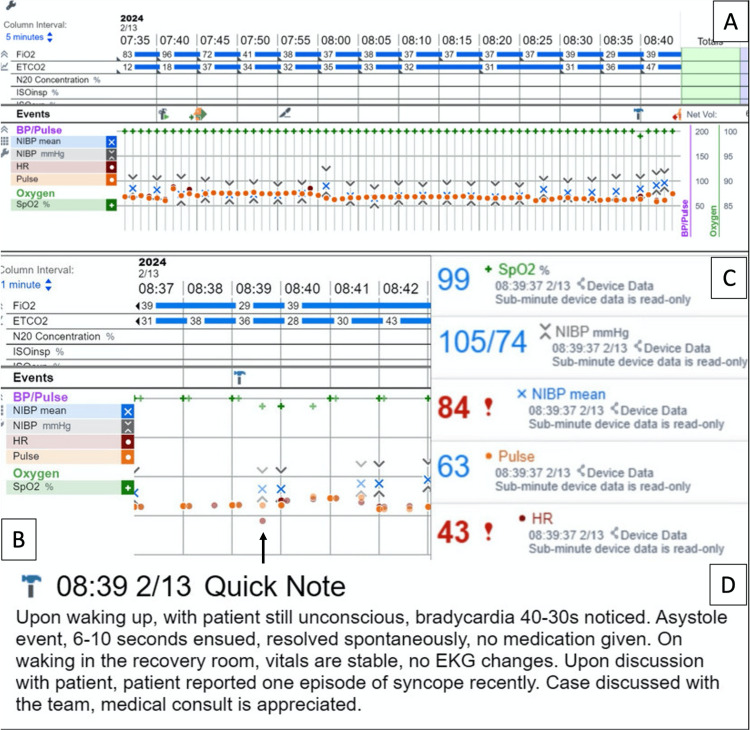
Intraoperative monitoring data A: The SpO2, BP, and ETCO2 monitoring graph every five minutes for the whole procedure. B: The monitor graph for every minute around the time of the incident shows a significant drop in HR recorded at around 08:39 from baseline (black arrow). However, asystole was not recorded due to the transient course. C: The monitoring data at 08:39 showed an HR of 43 bpm compared with baseline 60s. D: A 'quick note' of the incident. SpO2: Pulse oxygen saturation, BP: Blood pressure, ETCO2: End-tidal carbon dioxide, HR: Heart rate, bpm: Beats per minute

In the operating room, the patient was monitored by electrocardiogram, pulse oxygen saturation (SpO2), non-invasive blood pressure (BP), and end-tidal carbon dioxide (ETCO2) (Figure 1). Figure 1A displays monitoring data every five minutes. Figure 1B shows the monitoring data at a one-minute interval around the time of the event (08:39). Figure 1C demonstrates a heart rate (HR) of 43 beats per minute (bpm) captured at 08:39; however, asystole was not recorded due to its transient nature. Figure 1D shows the quick event note. 

General anesthesia was induced with midazolam 2 mg, fentanyl 50 mcg, and propofol 200 mg. A laryngeal mask airway (LMA; I-gel size 4) was inserted with satisfactory tidal volume and airway pressure. General anesthesia was maintained with sevoflurane at approximately 2.5%, with fentanyl 50 mcg administered at the beginning of the surgery. The surgery was uneventful, with vital signs stable throughout, 600 ml of lactate ringers infused, and minimal blood loss. The patient was in the Trendelenburg position during the procedure and was placed in the supine position during emergence. After several minutes, the patient was not breathing spontaneously, so a jaw thrust was applied to stimulate breathing and facilitate a quicker emergence. Shortly after the application of the jaw thrust, the HR precipitously dropped from the 70s to the 40s within seconds. Before the administration of atropine, the patient developed asystole. The jaw thrust maneuver was immediately discontinued, and code blue was activated. Seconds later, the patient's sinus rhythm spontaneously resumed, achieving the return of spontaneous circulation (ROSC) without atropine. To confirm it was caused by the jaw thrust maneuver, the anesthesia team repeated the maneuver briefly, and severe bradycardia ensued shortly. With the cessation of the maneuver, normal sinus rhythm resumed almost immediately. Due to the transient nature of the course, HR and BP changes were not reflected on the chart.

**Figure 2:**
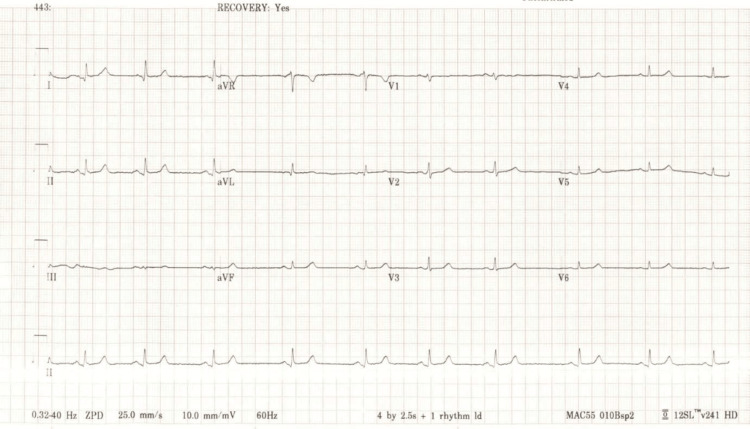
The first EKG done one hour after surgery shows sinus bradycardia and arrhythmia, likely the cardiac consequence of prior asystole.

**Figure 3:**
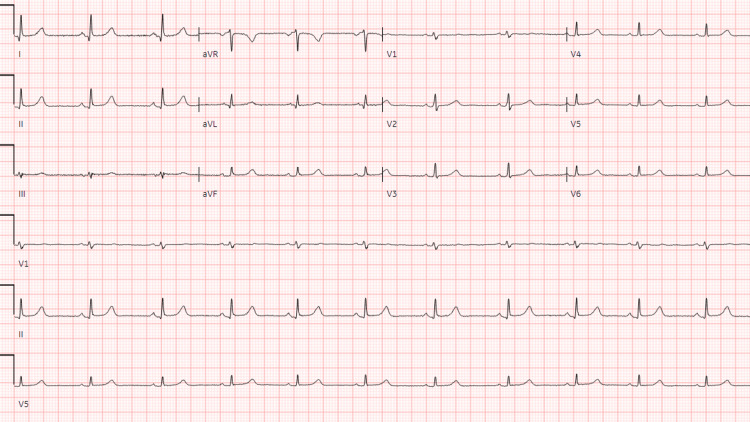
The second EKG done 1.5 hours after surgery shows normal sinus rhythm, and no arrhythmias.

Vital signs remained stable throughout the rest of the emergence. The patient was awake, alert, and oriented and was transported to the post-anesthesia care unit for recovery. Subsequent laboratory workups were within normal limits, including troponin (<6 ng/mL for three times with a five-hour interval), thyroid-stimulating hormone (TSH, 1.95 uIU/mL), and N-terminal pro-b-type natriuretic peptide (NT-proBNP, 51.5 pg/mL). The first EKG was done one hour after the surgery (Figure 2), which showed sinus bradycardia and sinus arrhythmia. The second EKG 30 minutes later showed normal sinus rhythm (Figure 3). Telemetry monitoring later in the medical ward showed no arrhythmias. The echocardiogram revealed no cardiac structural abnormalities. The patient was discharged safely and had no relevant complaints during her follow-up appointments six months postoperatively.

## Discussion

This case is known to be one of the first case reports of jaw thrust-induced asystole. Our patient developed profound transient bradycardia and asystole when the jaw thrust maneuver was applied. The first EKG revealed bradycardia and sinus arrhythmia, which is most likely the residual manifestation after ROSC following asystole. The second EKG, telemetry, and echocardiogram findings suggest that the patient does not have underlying significant cardiac disease. Relevant labs were within normal limits. Based on the results above, we deemed the mechanism behind this complication to be the vasovagal response from the intense pain caused by the application of the jaw thrust. This case highlights the intricate and noteworthy relationship between the autonomic nervous system and the cardiovascular system.

Vasovagal syncope occurs when a sudden increase in vagally-mediated parasympathetic tone decreases HR. It is one of the most common causes of transient syncope during anesthesia, which typically has a benign clinical course and recovers spontaneously. However, asystole and circulatory collapse can appear and result in permanent brain damage. Precipitating factors may be sudden pain [3], surgical manipulation, or trauma. In our case, it is postulated that profound parasympathetic neuron activation elicited by muscle and joint stimulation and cranial V activation, as well as many other perioperative factors, have contributed to the clinical manifestation.

It has been reported that pain originating in deep structures such as joints and muscles may evoke profound decreases in BP and HR by preferentially activating the ventrolateral periaqueductal gray matter (vlPAG), evoking a conservative/withdrawal response characterized by a decrease in BP and HR [4]. In contrast, cutaneous pain activates the lateral periaqueductal gray matter (lPAG) within the midbrain and evokes an excitatory flight/fight response characterized by increases in BP and HR. First, in this case, the jaw thrust maneuver involves pain stimulation on multiple jaw muscles, including the masseter, temporalis, medial pterygoid, and lateral pterygoid, as well as the temporomandibular joint (TMJ). The profound stimulation of the muscle groups and joints has a significant contribution to the vasovagal response. 

Second, the jaw thrust procedure stimulates the fifth cranial nerve and causes trigeminal cardiac reflex (TCR). This brainstem reflex manifests as typical hemodynamic perturbations, including sudden lowering of HR and asystole. The proposed mechanism of this reflex is sensory stimulation of the trigeminal nerve through the gasserian ganglion to the sensory nucleus of the trigeminal nerve, which then connects to the nucleus ambiguous and dorsal motor nucleus of the vagus, which then activates the cardioinhibitory efferent fibers to the myocardium [5]. Current reports are mainly about surgical procedures, especially clinic dental cases [6]. In our case, the patient underwent vigorous mandible manipulation and trigeminal overstimulation, which may have promoted the parasympathetic response.

Third, for patients who are scheduled for surgery and anesthesia, the physical and psychological stimulation and the stress can be triggers for vasovagal syncope. Other comprehensive perioperative factors, including the procedure (hysteroscopy is known to activate the parasympathetic nervous system by manipulating the cervix or uterine cavity [7]), perioperative anxiety, dehydration, and baseline hypotension [8], may have all contributed to the significant vasovagal syncope episode. 

Park et al. reported on the contradictory effects of a jaw thrust on HR, showing increased sympathetic activity during the jaw thrust maneuver [9]. Fazalbhoy et al. demonstrated a dichotomy of responses in muscle sympathetic nerve activity (excitatory response vs. depressed activity), which could be explained by the individual difference in the degree of sympathetic and parasympathetic control over the heart, known as the low-frequency band (LF)/high-frequency band (HF) ratio as an index of the sympathovagal balance [10]. This may explain the opposite findings on performing the maneuver. Management includes timely cessation of triggers, the Trendelenburg position, glycopyrrolate, or atropine. After vasovagal episodes, close observation as well as cardiac and neurology consults may be needed. 

## Conclusions

The authors experienced a case where a young, healthy female undergoing hysteroscopy developed a transient yet severe vasovagal syncope from the jaw thrust procedure. Possible mechanisms may include pain from deep muscle and joint stimulation and trigeminal nerve activation, as well as other predisposing factors, including surgery type and patient factors such as dehydration, anxiety, and baseline hypotension. Under general anesthesia, typical symptoms are absent, like nausea, dizziness, and blurred vision. The initial and only presentation can be sudden onset bradycardia and asystole. This case report emphasizes the significance of being vigilant for EKG changes when performing the jaw thrust maneuver. Identifying the risk factors and preventing and treating vasovagal reactions is critically important for anesthesiologists.
